# Iodine in the Treatment of Dental Periostitis and Diseases of the Gums

**Published:** 1851-07

**Authors:** James North

**Affiliations:** Bangor, Me.


### ARTICLE III.

### *Iodine in the Treatment of Dental Periostitis and Diseases of the Gums.* By JAMES NORTH, M. D., D. D. S., *Bangor, Me.*

I SHALL introduce as the subject of the few remarks that I shall offer on the present occasion, the history and treatment of several cases, which may serve to illustrate the beneficial action of iodine, in diseases of the teeth and gums.

In the month of August, 1843, I was desired to visit a Mrs. Copland, of Pittsfield, on account of a harmaturia, accompanied with pain in the region of the kidneys She informed me, that her health previous to the last year had been good. In April, 1842, she was prostrated with pneumonia, which she barely escaped with life. From effects, consequent of the treatment of this disease, she dates the origin of her present troubles. Soon after a partial recovery, she was afflicted with neuralgia of the facial nerve, of the seventh and the second branch of the fifth pair of nerves, on the left side of the face. In the early stage of the disease, she had submitted to the extraction of all the teeth in the left side of the upper jaw, to the division of the nerve, to the operation of alteratives, opiates and tonics, without mitigation of pain or amendment of the disease. In the early part of 1843, she was occasionally attacked with pain in the left lumbar region, of the same character, and occurring at the same hours, with the pain in the face. Accompanying the lumbar pain were other symptoms, indicating serious functional derangement of the kidneys. On the morning of the 22d of August, after evacuating the bladder, she was surprised with fear, to find the contents of the vessel, to be nearly all blood. For this last symptom I was called to prescribe. At this time Mrs. C. was in the 45th year of her age, and of a nervous and scrofulous constitution, she had a care-worn and haggard countenance, indicative of anxiety and long suffering ; a fetid breath, caused by carious teeth and ulcerated gums. Her tongue was coated and dry, her pulse weak, frequent and intermittent. The usual remedies for arresting a tonic hemorrhage, were given without any benefit, other than improving the condition of the digestive organs. I now prescribed the iodide of starch, in combination with quinine, every six hours. I commenced with ten grains of the iodide, combined with one grain of quinine, and gradually increased the iodide to one drachm, and the quinine to five grains. I continued this treatment for three weeks, at the end of which time the hemorrhage had ceased, and the urine had assumed its natural color and properties. But the most important result of this treatment was in the improvement and final cure of the neuralgia. This disease had exhibited many of the worst symptoms of the disease designated as tic douloureux. It was confined to the left side of the face in the nerves distributed over the cheek bone, just below the orbit, the upper left teeth and gums, and also in the ear and mastoid process. She had been under the care of Dr. George Gourley of Morridgwork, an ingenious and skilful physician. He had given large doses of carbonate of iron, Fowler's solution of arsenic, quinine, morphia and belladonna. Externally he had used stimulating and anodyne embrocations, blisters, topical bleeding by means of leeches, antimonial and mercurial ointment, and the ointment of veratria. She submitted to this course of dragging and torturing, with the heroism of a martyr, and without reaping the least permanent benefit. Again and again disappointed in her hope of recovery, losing confidence in her physician, and disgusted with his prescriptions, she abandoned her case to the course of nature. This was three months previous to my first visit. After prescribing the iodine for three weeks, the constitutional improvement was so evident, Mrs. C. was very anxious to continue its use; at the end of two months it was discontinued. The neuralgia was cured, the gums were healthy, the countenance assumed a healthy look, and all the functions were in the performance of their legitimate action. Early in the month of January, 1844, I was again summoned to visit Mrs. Copland. I found her dejected and sorrowful; and was informed that the neuralgia had returned. This time it was located in the left superior maxillary bone, and occurring at 6 o'clock in the morning, and passing off between 10 and 12, A. M. Aware of the unyielding character of the disease, I was not willing to trust those agents that had so signally failed in the first attack; consequently I commenced my treatment with the iodide of starch, combined with quinine, as before. The practice was successful; in three weeks she was discharged from my attendance, without neuralgia. I have often seen Mrs. C., and am happy to say, there has been no recurrence of the disease. Soon after the successful termination of Mrs. Copland's case, I was called to see a Miss Marstin, aged 18 years, for the purpose of extracting a painful tooth; two months since she had partially recovered from a typhoid fever. During the continuance of the fever, she took large doses of calomel; causing extensive ulcerations of the mouth and gums. She had a chlorotic countenance, her digestion was imperfect, costive bowels, together with the suppression of the catamenia; on examining the mouth, I found the tongue loaded with a dark moist coat; the gums were swelled, spongy and ulcerated. The second molar of the right lower jaw, was the tooth selected for extraction; it was not carious, but tender when touched, the pain was of a periodical character, she had been afflicted with it three weeks preceding my visit. I explained to Miss M. my opinion of her case, and advised a careful regimen, occasional laxatives and quinine, combined with iodine, as in the case of Mrs. Copland. The pain was relieved immediately, and after a continuance of the prescription for a month, she was restored to her usual state of health.

Rheumatic periostitis is a disease of common occurrence, and when the cause can be traced to the excitement of a diseased fang, or a cold, can readily be cured, by extracting the offending tooth, or the administration of simple remedies. But there are cases that present many embarrassments, both as regards diagnosis and treatment. The following case may serve to illustrate this position.

In April, 1844, I was requested to visit a Mrs. Foss, on account of a painful inflammation of the right lower jaw. The disease made its appearance in the early part of November, 1843, without any assignable cause. There was a pulsating and constant pain surrounding the first molar tooth, of this side, and after a few days, a secretion of pus. Her physician thought this might be the exciting cause of the disease, and it was extracted. No relief being afforded by the operation, on further examination, it was thought advisable to extract the remaining molar teeth. This measure was attended with no better result than the first extraction. She now applied to another physician, suspecting constitutional vice as the remote cause of the disease, he advised constitutional remedies. She was put upon an alterative and tonic course. The gums were leeched, and blisters applied externally over the region of the disease. This course was continued for a few weeks, but the disease proving obstinate, was abandoned. Soon after she applied for my advice. I found the gums inflamed and indurated, and the surrounding parts implicated in the diseased action. The tonsil, submaxillary and parotid glands were enlarged, hard and painful. The lower jaw was nearly closed, and deglutition difficult and attended with pain. I supposed the disease to be of a rheumatic origin, and its obstinacy to be dependent upon scrofulous or mercurial taint. I prescribed a judicious diet, occasional purgatives and the iodide of potash, in combination with syrup of sarsaparilla. To the surprise of myself and patient, the disease soon lost its painful character, and in less than a month it was completely eradicated.

From the history of the above cases, and others of a like character, that have occurred in my practice, I have come to the conclusion, that for the cure of neuralgia in persons of a scrofulous habit, or predisposed to it, or occurring in persons broken down by an abuse of mercury, there are no remedial agents so effectual as iodine, in combination with a tonic; I prefer the iodide of starch, from the fact, that it can be given in larger doses than in any other combination of this agent; it is not disagreeable to the taste, and can be administered in union with quinine, without injuring the properties of either. And in rheumatic diseases, acute or chronic, after proper depletory measures, I have rarely been disappointed in effecting a cure with the iodide of potash.

